# Flanking signal and mature peptide residues influence signal peptide cleavage

**DOI:** 10.1186/1471-2105-9-S12-S15

**Published:** 2008-12-12

**Authors:** Khar Heng Choo, Shoba Ranganathan

**Affiliations:** 1 Department of Biochemistry, Yong Loo Lin School of Medicine, National University of Singapore, 117597, Singapore; 2 Department of Chemistry and Biomolecular Sciences & ARC Centre of Excellence in Bioinformatics, Macquarie University, Sydney NSW 2109, Australia

## Abstract

**Background:**

Signal peptides (SPs) mediate the targeting of secretory precursor proteins to the correct subcellular compartments in prokaryotes and eukaryotes. Identifying these transient peptides is crucial to the medical, food and beverage and biotechnology industries yet our understanding of these peptides remains limited. This paper examines the most common type of signal peptides cleavable by the endoprotease signal peptidase I (SPase I), and the residues flanking the cleavage sites of three groups of signal peptide sequences, namely (i) eukaryotes (Euk) (ii) Gram-positive (Gram+) bacteria, and (iii) Gram-negative (Gram-) bacteria.

**Results:**

In this study, 2352 secretory peptide sequences from a variety of organisms with amino-terminal SPs are extracted from the manually curated SPdb database for analysis based on physicochemical properties such as p*I*, aliphatic index, GRAVY score, hydrophobicity, net charge and position-specific residue preferences. Our findings show that the three groups share several similarities in general, but they display distinctive features upon examination in terms of their amino acid compositions and frequencies, and various physico-chemical properties. Thus, analysis or prediction of their sequences should be separated and treated as distinct groups.

**Conclusion:**

We conclude that the peptide segment recognized by SPase I extends to the start of the mature protein to a limited extent, upon our survey of the amino acid residues surrounding the cleavage processing site. These flanking residues possibly influence the cleavage processing and contribute to non-canonical cleavage sites. Our findings are applicable in defining more accurate prediction tools for recognition and identification of cleavage site of SPs.

## Background

Amino-terminal signal peptides (SPs) [1,2] mediate the transport of prokaryotic and eukaryotic secretory proteins to the cell membrane and endoplasmic reticulum respectively. Synthesized as part of secretory precursor proteins (preproteins), SPs guide the preproteins to the targeted destination before being excised by the membrane-bound type I signal peptidases (SPase I) [3] during translocation across the cell membrane.

These transient "zip codes" measure between 13 to 36 amino acid residues (aa) [1] comprising a tripartite structure, with a central hydrophobic region, flanked by the amino and carboxy segments of the signal peptide. The "h-region" forming the hydrophobic core at the centre of the SP is lined with stretches of hydrophobic residues, said to adopt an α-helical conformation [4]. The length of the hydrophobic core varies with the organism. Preceding the hydrophobic core is the "n-region" containing positively-charged and polar residues, while the "c-region" at the end of the signal peptide adopts an extended β-conformation to facilitate recognition by SPase I. Other characteristic features have been described in detail elsewhere [2,5]. Apart from targeting, SPs have been reported to exhibit regulatory function in immune surveillance [6], to promote effective translocation by preventing the premature or misfolding of secretory preproteins [7], to control the amount of proteins to their destination [8,9] and possibly other functions as more revelations surface over time. Numerous studies [10-12] have also highlighted the adverse effects caused by mutation to SPs.

The functional repertoire of SPs warrants further investigation of their properties and their neighboring residues to advance our understanding of SPs for their crucial roles in the secretory pathways of both prokaryotes and eukaryotes [5,13].

The recent deluge of protein sequences have spurred the development of myriad computational tools and techniques [14-19] to predict the SP cleavage site. While the prediction accuracies of these tools vary depending on the datasets employed in their studies, they have generally achieved high levels of accuracy. Nonetheless, the precise mechanism governing the cleavage of the preprotein thus far remains a conundrum and the accuracy of even the best prediction methods for modifications to the signal peptide region remains unpredictable. As a means to understand the cleavage processing and the targeting mechanism, it is necessary to understand the intricacies of protein secretion, which include its SP and mature peptide (MP) moieties. An early study of 118 eukaryotic and 32 prokaryotic sequences conducted by von Heijne [20] provided excellent insights into the nuances of the differences between eukaryotic and bacterial SPs. Subsequent studies [21-23] investigated SPs and MPs, either singularly or in combination, often through gene fusion and mutagenesis studies to observe their translocation and differential expression levels. Wide-ranging studies [23-30] were conducted to inspect the charge bias, hydrophobicity and various aspects related to the physical chemical properties of SPs. Other studies examining the structural aspects of SPase I-substrate complexes through 3D-structures and computational models [31-34] were also carried out to study the substrate specificity of the cleavage site and the characteristics of the amino acid residues around the cleavage site. With the massive increase in protein sequences deposited to the public sequence databases since 1999, there is a tremendous opportunity to further explore our understanding of SPs and their mechanisms.

In this respect, we have extracted an updated, manually curated set of 2352 eukaryotic and bacterial SPs [described in **Methods**] to examine the characteristics of the amino acid residues at the cleavage site, representing an updated large-scale, comprehensive analysis of SPs, based on manually curated data. Furthermore, we have carefully analysed the residues composition in the vicinity of the cleavage site, as a multitude of site-directed mutagenesis studies have revealed that residues upstream and downstream of this site affect cleavage processing [25,35].

## Results

### Ensuring quality of the dataset

Initiating this study with a high quality dataset is crucial. In this study, we have restricted our investigation to 2352 secretory sequences containing amino-terminal SPs. The curated dataset is available from Additional file 1.

During our manual investigation phase, where we plotted scatter plots of the assembled SPs, *β*-hexosaminidase A [Swiss-Prot: HEXA_PSEO7], an *αβ*-subunit heterodimer lysosomal hydrolase was identified as an outlier. Tsujibo *et al. *[36] indicated that the SP cleavage site is 11 aa and added that its SP does not possess the typical tripartite features of an SP. However, sequence comparison against other species using Swiss-Prot database reveals lengths of approximately 18 to 22 aa. Due to this inconsistency, this entry was manually removed from the final dataset.

### Examining eukaryotic and bacterial datasets

**Figure 1:**
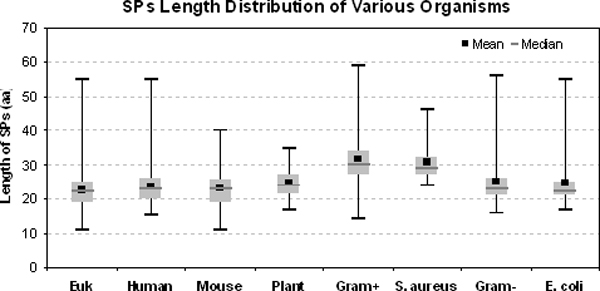
**Boxplot illustrating the SPs distribution found in selected organisms and groups (Eukaryotes, Gram-positive and Gram-negative bacteria).** Mean length (■) and median (-, grey bar) values are indicated.

**Figure 2:**
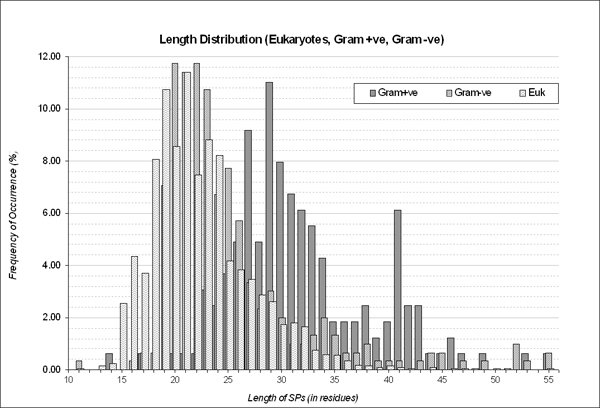
**Signal peptides from the three organism groups measured based on their length.** The Y-axis shows the frequency of occurrences for a specific length of signal peptide while the X-axis depicts the various lengths.

The cleansed data was grouped into (i) eukaryotes (Euk) with 1877 sequences (ii) Gram-positive (Gram+) bacteria with 168 sequences and (iii) Gram-negative (Gram-) bacteria with 307 sequences. From the boxplot (Figure 1), SPs of Gram+ (SPs_Gram+_) tend to be longer with median length of 30 aa and display a bi-modal distribution with peaks at 29 aa and 41 aa (Figure 2) as compared to SPs of Euk (SPs_Euk_) and SPs of Gram- (SPs_Gram-_) which carry median length of 22 aa and 23 aa respectively. Interestingly, SPs_Euk _and SPs_Gram- _exhibit somewhat similar SP length distribution although 4.5% or 14 SPs_Gram- _extend beyond 40 aa. In spite of the wide range of SP lengths permissible within many groups of organisms excluding SPs of plants (SPs_Plant_), the majority of the lengths within the groups still fall in the 25th to 75th percentile, affirming the many studies which have reported SPs as having variable length.

**Figure 3:**
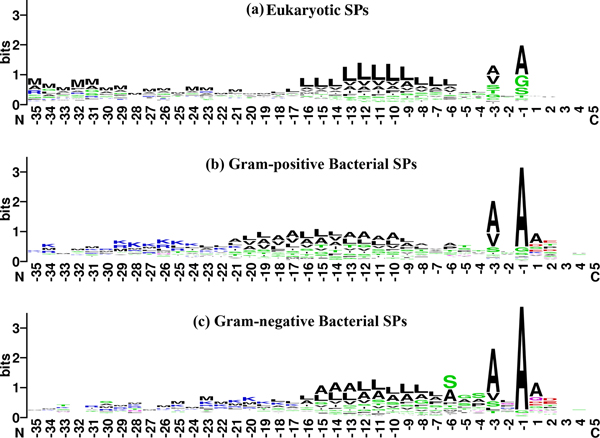
**Sequencelogos 37 of eukaryotic and bacterial (Gram-positive and Gram-negative) signal and mature peptides starting from posistion -35 to +5.**[[Bibr B37]] The interface between position -1/+1 represents the SPase I cleavage site. The amino-acid residues are grouped and coloured based on the R group of their side-chain. Red denotes polar acidic amino-acid residues (D, E); Blue denotes polar basic amino-acid residues (K, R, H); Green denotes polar uncharged amino-acid residues (C, G, N, Q, S, T, Y); Black denotes non-polar hydrophobic amino-acid residues (A, F, I, L, M, P, V, W).

The cleavage site, designated P1–P1', occurs between residues located at position -1 (the last residue of the SP or P1, prior to the scissile peptide bond) and +1 (the first residue of the MP or P1'). Figure 3 depicts the sequence logos [37] for the three groups starting from position -35 (P35) to position +5 (P5'), spanning contiguous segments from the SP and MP moieties.

P1 and P3 favour small, aliphatic residues; in particular Ala and Val, which inclination is strikingly apparent in bacterial SPs. Glycine (Gly), serine (Ser) and threonine (Thr) are also noticeable at these two positions in SPs_Euk_. P2 of SPs_Euk _exhibits preferences for Leu (15.2%) and Ser (12.0%) whereas different sets of amino acids: {Ser (12.5%), glutamine (Gln) (11.9%), phenlyalanine (Phe) (11.9%), Ala (11.3%)} and {Leu (17.6%), Gln (14.3%), Phe (11.4%), His (11.4%)} are preferred by SPs_Gram+ _and SPs_Gram- _respectively [see Additional file 2 for the frequency matrices]. From P1' onwards, there is no obvious pattern of amino acid conservation in SPs_Euk _with the exception of slightly enhanced occurrences of Ala (13.5%) and Gln (11.0%) at P1'.

Compared to eukaryotic SPs, the amino acid composition is different in bacterial SPs. In the case of SPs_Gram+_, P1' is mostly occupied by Ala (36.3%), Asp (11.3%), Ser (10.7%) and Glu (9.5%). P2' is populated by Thr (14.3%), Glu (13.7%), proline (Pro) (13.1%), Ser (10.7%) and Asp (10.7%). Lys (13.1%) is the dominant amino acid at P3' while Pro (14.3%) and Thr (14.3%) are preferred at P4'. Beyond P4', there are no clear patterns if we were to compare the relative frequencies between the adjacent positions for the same amino-acid type. Similarly for SPs_Gram-_, P1' is populated by Ala (41.7%), Gln (12.1%), Asp (7.2%) and Glu (6.2%) whereas P2' is largely distributed between Asp (17.3%), Glu (16.9%), Pro (10.8%) and Thr (10.8%). From P3' onwards, when we compared the relative frequencies of each amino acid with respect to its adjacent positions and also within the column [see Additional file 2] and (Figure 3), we could not ascertain any discernible patterns. His, tryptophan (Trp) and tyrosine (Tyr) are clearly under-represented in all three groups of SPs and for all the positions (P10 to P10') that we examined while Cysteine (Cys) is almost nonexistent in bacterial SPs throughout the aforesaid positions. Pro is visibly avoided in positions from P3 to P1' but relatively prevalent at P4 and P2'. In contrast, Gly, Ile, Thr (except at P1 in bacterial SPs), Val (except at P1), Ser and particularly Ala (especially at P3, P1 and P1') are ubiquitous in all the positions that we profiled.

In all three groups of SPs, acidic residues (Asp and Glu) are pronounced from P1' onwards. Similar trends can be seen for basic or positive-charged residues comprising Arg, Lys and His. In fact, when we group the basic and acidic residues (see Additional file 2], we observe consistent and modest occurrence of these charged residues across all three groups of SPs from P1' onwards, inclusive of P2 but conspicuously absent or appearing in minute amounts at P3 and P1, most prominently in the eukaryotic MPs. Basic residues, Arg and Lys are common at the n-region of bacterial SPs.

**Figure 4:**
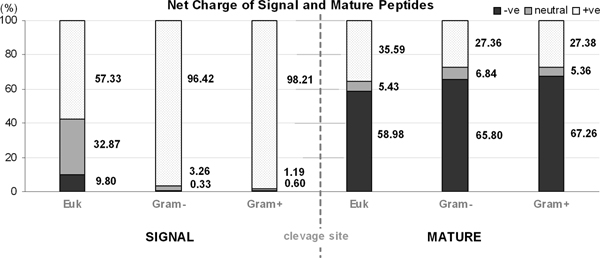
**Net charge calculations of signal and mature peptides for the three groups of organisms.** The net charges are grouped into three classes: positive (>0), neutral (= 0) and negative (<0) charge. The numbers represent the frequencies of which the charges are observed.

Interestingly, when we measure the net charge of SPs and MPs individually (Figure 4), bacterial SPs are overwhelmingly positive-charged (>0) while their MPs gravitate towards a net negative-charge bias. Median net charge for SPs_Gram+ _and SPs_Gram- _are +3 and +2 respectively. Eukaryotes share a somewhat similar net charge distribution in their MPs when compared to MPs_Bacteria _but their SP moieties support a more uniform net charge distribution (+ve: 57.3%; neutral: 32.9%; -ve: 9.8%) in comparison to the positive-charge preference in SPs_Bacteria_.

**Figure 5:**
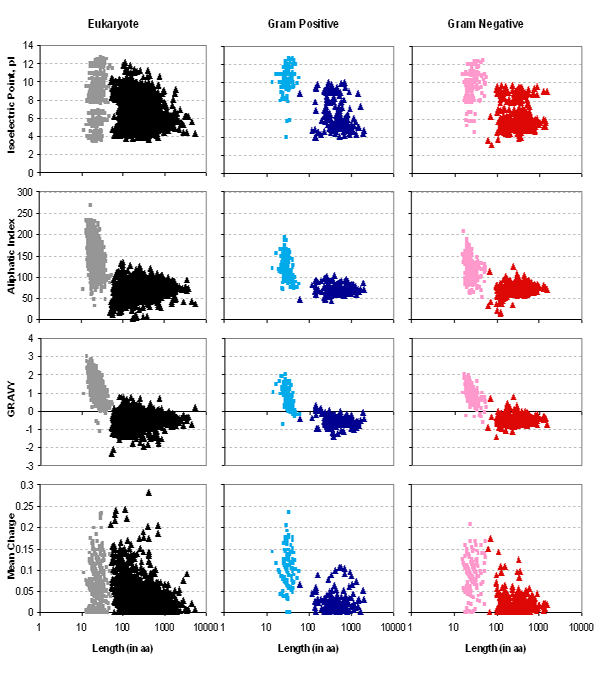
**Comparison of the isoelectric point (pI), aliphatic index, GRAVY value and mean charge among the three organism groups.** Data are represented by squares which denote SP while triangles denote MP.

To examine the extent of differences in amino acid composition between the SP and MP moieties of eukaryotes and bacteria, we constructed scatter plots (Figure 5) of isoelectric point (p*I*), aliphaticity, GRAVY and mean charge calculations plotted against the length of SPs (■) and the corresponding MPs (▲). In all three groups of organisms, we observed that the overall computed values of MPs tend to be clustered in a narrower range when compared with SPs. For instance, based on the calculation using the aliphatic index, MPs_Gram+ _lie mostly between 50 to 100 within the scale whereas SPs_Gram+ _occur anywhere between 75 to 200. A similar trend such as this exists in the other calculations including GRAVY and p*I *except for the p*I *of MPs_Euk_. SPs_Euk _form two clusters based on p*I *calculation whilst SPs_Gram+ _and SPs_Gram- _are predominantly represented within single clusters with median p*I *values of of 10.3 and 10.0, respectively. From hydropathicity calculations, the GRAVY score of SPs are largely positive (SPs_Euk_:99.7%; SPs_Gram+_:93.5%; SPs_Gram-_:97.7%) indicating a hydrophobic propensity. MPs, on the other hand, show preferences towards hydrophilic nature (MPs_Euk_: 93.7%; MPs_Gram+_: 94.6%; MPs_Gram-_: 95.1%).

## Discussion

The aim of this study is to uncover details about SPs, based on their primary structure, to understand the possible correlations with their structure, variability in length and composition and any distinct features around the cleavage processing site. Therefore, we have included the MP moiety in addition to the SP, since exploring the environs of the scissile bond may provide clues to the hitherto reported features of SPs.

### Inter-group differences

Our results indicate that SPs_Gram+ _and SPs_Gram- _share more similarities, compared to SPs_Euk_. When we measured the net charge of the SP moieties of these three groups (Figure 4), we observe that SPs_Euk _is distinctly different from the bacterial SPs in that bacterial SPs overwhelmingly favour a net positive charge bias whereas SPs_Euk _do not exhibit any such inclination. Moreover, from the constructed frequency occurrence matrices (shown in Additional file 2) as well as the sequence logos (Figure 3) of these three groups, it becomes clear that the bacterial datasets bear much resemblance in their overall features and properties, such as the diverse variability in their SPs primary structure, the highly-visible P3-P1 sequence motif which exhibits high selectivity for small, aliphatic residues and a detectable hydrophobic-region (h-region) at the core of SPs. Even so, underlying these commonalities are inter-group differences, albeit subtle in some cases. For example, mean length and h-region of SPs_Gram+ _are considerably longer than those of SPs_Gram- _and SPs_Euk_. In the case of the tripartite structure consisting of n-region (positively charged), h-region (hydrophobic) and c-region (neutral and polar) which are commonly reported in the literature, our findings show that this structure is pronounced in the bacterial SPs but somewhat ambiguous in SPs_Euk_, specifically in the n-region where positively-charged residues are far less prominent. Likewise, the sequence motif at P3 and P1 of bacterial SPs is almost dominated by Ala and Val, while such exclusivity is not asserted in SPs_Euk _where a number of other different amino acids are tolerated. These nuances are likely attributed to the differences in their cell-membrane structures, suggesting certain overall, minimal requirements at the sequence and possibly at structure level [38] as well that a SP must conform to, for recognition and processing in the secretion pathway. Perhaps this may account for the seemingly contrasting selectivity for certain types of amino acids at certain subsites while simultaneously maintaining a generous accommodation for amino acid degeneracy at other subsites in the SP.

### Influences of the mature peptide moiety

Since the (-3, -1) rule [39] was proposed, where small, uncharged residues are favoured at the P3 and P1 positions, the SP moiety has drawn much attention. A fair number of ensuing reports [22,25,40-43] began to explore the influences of the MP moiety besides the SP and many such studies continue to furnish additional support and evidence to advance our comprehension of the less understood role of the amino acids at the MP moiety. Numerous studies [40,44] experimented with SPs by fusing them to an assortment of secretory and non-secretory proteins for homologous and heterologous secretion and demonstrated that the SP alone is not sufficient to ensure the processing of secretory proteins, implying that a section of the MP must contribute to the process. In fact, such studies have shown that a balance between the SP and portion of the MP moiety affects export efficiency [45-47].

When we examine the frequencies between the adjacent positions of ten amino acid residues from both sides of the cleavage site (data shown in Additional file 2) *viz. *SP (P10-P1) and MP (P1' – P10') for all three organism groups, the frequencies of charged residues (counting both positively and negatively charged residues) are relatively stable. The transition value from one position to another does not fluctuate beyond 50% of the difference for the MP moiety. For the SP moiety (P10-P1), the fluctuations are more dramatic at P5, P4 and P2 (although less pronounced for gram-negative bacteria) while virtually absent at other positions. When we divided the charged residues into positively and negatively charged subgroups, we observed that a specific charged subgroup is preferred at certain positions. Moreover, when we measured the mean charge using a sliding window of variable size (3 to 11; data not shown), we noticed that the fluctuations between the positively and negatively charged residues seem to converge and stabilize at around P8' to P10' whereas uncharged residues maintain a uniform trend throughout all the positions.

Approximately a quarter of the bacterial MPs and 35% of MPs_Euk _bear a net positive charge, 5–6% are neutral while the majority of MPs favour a net negative charge. This is in stark contrast to the SP moiety which is inclined towards a net positive charge, the trend being especially strong in bacteria. Probably, secretory proteins maintain their desired net charge levels within the SP and MP moieties to enable their interaction with other players in the secretion pathway. This can be done by varying or accommodating diverse amino acids at selected positions while being rigid in the choice of amino acids at others. This selectivity is visible at some MP positions particularly those in the vicinity of the cleavage site but not further downstream.

Kajava et al. [25] proposed that a net charge with null or negative bias should be maintained for the first 18 amino acid residues of the MP, to promote successful expression of proteins in Gram-bacteria and any optimization performed on the SP should include the specified region. However, we do not observe any significant pattern beyond P5' at the MP moiety based on our results (Figure 1, Figure 3 and Additional file 2) to support this proposal, possibly because the first 18 residues could include several combinations of SP and MP moieties. Moreover, if we compare the relative frequencies of adjacent positions at the MP moieties, they appear to be rather stable. Our results are in general agreement with other studies that include the MP moiety, but the extent of the region to be included remains debatable. The varying results from the different studies make it difficult to compare and obtain consensus. Furthermore, the paucity of crystal structures solved to date (only three SPase I-related entries are found in Protein Data Bank [48]) adds to the challenge of deciphering the extent of MP involvement in the secretory pathway.

### Recognition of the cleavage site and its flanking region

From our dataset, out of 1877 eukaryotic, 168 gram-positive and 307 gram-negative sequences, the occurrence frequencies of the consensus sequence motif Ala-x-Ala at P3 and P1 are 14.5%, 47.0% and 58.9% respectively. This is much lower than the frequencies for the individual position columns of Ala (Additional file 2), implying that the sampling space for cleavage site recognition is not limited to the Ala-x-Ala motif. In our previous study [31] where we modeled the 3D-structure of *E. coli *SPase I substrate-complex using computational approach, our model suggested that amino acid residues upstream and downstream of the cleavage site may influence substrate cleavage. The various subsites identified in that modeling study suggest amino acids of certain properties such as the nature, size and charge of the side-chain, can be accepted at these pockets. If we scrutinize these flanking residues further in the light of our current results, more significant patterns become prominent. Pro is implicated as a structure disruptor due to its steric hindrance from its cyclic side-chain and inability to form a hydrogen bond that stabilizes a helix [49]. Pro is often found at the end of α-helices, in turns or loops but produces a bend when it appears in the middle of an α-helix. Pro is markedly disfavoured from P3 to P1' but it is comparatively prevalent at P4 and P2' (Additional file 2). The absence of Pro at these positions is consistent with reports on impaired function or inhibition of SPase I with Pro appearing at this position [50,51]. Glycine, another helix-breaking residue, is also spotted in modest amount at P5 and P4. Karamyshev *et al. *have shown that a β-turn is present at the P5 to P1 region of SPase-substrate complex [52]; our model [31] also generated a similar structure, which is consistent with the residue occurrence patterns in these positions (Additional file 2). The canonical Ala-x-Ala sequence motif for the SP cleavage site is only able to account for approximately half of the recognition sites. By considering these flanking residues, many non-canonical cleavage sites can be accounted for. These features working in concert provide the secretory machinery flexibility, versatility and perhaps accuracy to enact the signal peptide recognition processes.

## Conclusion

In this study, we have compiled a manually curated set of experimentally determined amino-terminal SP-containing sequences and analysed the cleavage sites and flanking regions of three organism groups namely eukaryote and bacteria (gram-positive and gram-negative). Our findings show that the three groups share several similarities in general, but display distinctive features upon examination in terms of their amino acid composition and frequency of residue occurrence, characterized by various physico-chemical properties. Thus, analysis or prediction of their sequences should be separated and treated as distinct groups. Further, we survey the amino acid residues surrounding the cleavage processing site and conclude that the domain recognized by the SPase I extends into MP to a limited extent. These flanking residues possibly influence the cleavage processing and constitute non-canonical cleavage sites.

Our large-scale analysis work uses substrate proteins derived from a variety of organisms and can help in defining more accurate prediction tools for the recognition of SPs and the identification of their cleavage sites. Our findings are also applicable to the design of more efficient SPs used in heterologous protein secretion.

## Methods

### Dataset manually curated and extracted from SPdb

We assembled a preliminary dataset containing 2512 sequences using the manually-curated Signal Peptide database (SPdb) Release 5.1 [53]. SPdb contains sequences which were reported with experimentally-verified SP cleavage sites as opposed to computational prediction, classified "putative" in several protein sequence and signal peptide data resources. The data in SPdb were extracted from the Swiss-Prot [54] Release 55.0 and EMBL [55] Release 93 sequence databases, based on a set of filtering criteria, described in detail elsewhere [53]. Viral and archaeal SPs were excluded as there were too few to render any meaningful analysis. Sequences that contain ambiguous positions or non-standard amino acids as identified by the characters 'X', 'Z' or 'U' found in their MP moiety were discarded. SPase II-cleaved lipoprotein SPs [56] and SPs of Twin-arginine translocation (Tat) proteins [57] were also deliberately omitted from this study since these SPs rely on different mechanism for processing their cleavage sites. In the process of assembling the dataset, we investigated the need for redundancy reduction [44,58] as we were concerned about the bias or over-representation of certain classes of sequences in the dataset. CD-HIT (version 3.1.1) [59] was used to cluster the sequences and removed sequences with sequence identities 100% in their SP moiety as studies [10-12,35] have shown that even a single substitution in amino acid could result in a pronounced effect.

The dataset was split into two sub-datasets based on the sequence moieties (i) SP and (ii) MP before being clustered with global sequence identity threshold set at 0.9; word size of 5 and other parameters assume the program's default. In each cluster, homologous sequences with 100% sequence identity in the SP moiety were discarded. Identical full-length (SP+MP) sequences were implicitly removed as a result. The reduced dataset of 2352 SPs-containing sequences were further categorized into three groups namely (a) Gram+ bacteria (*Firmicutes*, *Actinobacteria*, *Deinococcus*, *Fibrobacteres*, *Thermotogae*); (b) Gram- (*Proteobacteria*, *Spirochetes*, *Bacteroidetes*, *Cyanobacteria*, *Aquificae*, *Chlamydiae*) and (c) eukaryotes (see additional file 2) as the SPs of these three groups display distinct features [2,5]. Subsequently, we computed the physico-chemical properties of the SP and MP moieties for every sequence using ExPASy ProtPram [60]. The calculations include molecular weight, theoretical isoelectric point (pI), aliphatic index, GRand AVerage of hydropathY (GRAVY) and absolute mean charge.

### Calculations of the physicochemical properties

Size dimension is assumed to influence the bending of a peptide chain where the size of an amino acid is determined by the length and bulkiness of its side chain [24]. But since molecular weight (MW) of an amino acid is easier to measure and it is roughly proportional to its size, we thus use MW as an approximation.

pI is defined as the pH value where a given protein has no net charge and it often has the lowest solubility. Different algorithms exist to calculate pI rendering different values due to the different set of p*K*_*a *_values used. The p*K*_*a *_values adopted in this study were described by Bjellqvist *et al. *[61].

Aliphatic index [62] measures the relative volume occupied by aliphatic side chains (Ala, Val, Ile and Leu) of a protein according to the formula:

*AliphaticIndex *= *X*_*A *_+ a*X_*V *_+ b*(*X*_*I *_+ *X*_*L*_)

where X_A _(Ala), X_V _(Val), X_I _(Ile) and X_L _(Leu) are mole percent (100 * mole fraction) of the respective amino acid residue. The coefficients *a *and *b *are the relative volume of Val side chain (*a *= 2.9) and of Leu/Ile side chains (*b *= 3.9) compared to the side chain of Ala.

GRAVY [63] is an estimation of the overall hydrophobicity of a protein, but it does not take into account of interaction or positional effect of adjacent residues. Given a protein sequence *S*, its GRAVY score is computed as:

$$GRAVY(S)=\sum_{i=1}^{20}\alpha_{i}f_{i}$$

where *i *is one of the 20 standard amino acids; *f*_*i *_is the relative frequency of *i *in *S*; *α*_*i *_is the hydropathy value of *i *according to the scale propounded by Kyte and Doolittle [63] and *n *is the total number of residues in the sequence.

Net charge is the algebraic sum of all the charged amino-acid residues present in SPs and MPs calculated using the equation:

$$\text{Net Charge}=\sum_{i=1}^{20}\alpha_{i}f_{i}$$

The 20 standard amino acids are represented by *i *and *f*_*i *_represents the relative frequencies of occurrences of the amino acid *i*. Positively-charged residues (arginine (Arg), histidine (His) and lysine (Lys)) are assigned *α*_*i *_= 1 whereas negatively-charged residues (aspartic acid (Asp) and glutamic acid (Glu) are set as *α*_*i *_= -1. All other amino acid residues are assigned *α*_*i*_.= 0.

The *iep *program, part of the EMBOSS bioinformatics package (version 2.9.0) [64] was used to calculate the mean charge at neutral pH. The absolute value of the mean charge is further divided by the length of the polypeptide.

Mean hydrophobicity is defined as the arithmetic mean of the normalized hydrophobicity values of all the residues in the polypeptide where hydrophobicity was calculated using as defined by Kyte and Doolittle [63].

## Competing interests

The authors declare that they have no competing interests.

## Authors' contributions

KHC curated the dataset and conducted the analysis work; SR directed the study and both authors prepared the manuscript.

## Supplementary Material

Additional file 1 **Curated dataset used to perform this analysis**. 2352 secretory sequences containing amino-terminal SPs extracted and filtered from SPdb deposited into three worksheets, arranged according to the three organism groups namely eukaryotes, Gram-positive and Gram-negative bacteria. Each worksheet contains 7 columns of data namely *Entry_name*, *Description *(of the protein), *Organism*, *SP_Length *(length of the signal peptide), *Prot_length *(length of the protein sequence), *Signal Peptide *(signal peptide sequence), *Mature Peptide *(mature peptide starting from P1' and stops at the first 30 residues). Click here for file

Additional file 2 **Frequency matrix for eukaryotes and bacteria datasets**. Amino acid frequency matrix for the signal peptides and mature peptides of eukaryotes and bacteria. Percentage occupancy values from P10 to P10' [-10, +10] are shown, with the cleavage site in dotted line at -1/+1. Significant high and low values, in bold font are highlighted:grey: >10%; black: most preferred residue(s); cyan: charged residue group and green: aliphatic group. Click here for file
