# Supplementary material for: Flanking signal and mature peptide residues influence signal peptide cleavage
Source: BMC Bioinformatics. 2008 Dec 12;9(Suppl 12):S15. doi: 10.1186/1471-2105-9-S12-S15 (PMC2638155; doi:10.1186/1471-2105-9-S12-S15)
Supplement: Additional file 2 — Frequency matrix for eukaryotes and bacteria datasets. Amino acid frequency matrix for the signal peptides and mature peptides of eukaryotes and bacteria. Percentage occupancy values from P10 to P10' [-10, +10] are shown, with the cleavage site in dotted line at -1/+1. Significant high and low values, in bold font are highlighted:grey: >10%; black: most preferred residue(s); cyan: charged residue group and green: aliphatic group. [file 1471-2105-9-S12-S15-S2.pdf]

Flanking signal and mature peptide residues influence signal peptide cleavage by Khar Heng Choo and Shoba Ranganathan

Additional File 2. Amino acid frequency matrix for the SPs and MPs of eukaryotes and bacteria. Percentage occupancy values from P10 to P10’ [-10, +10] are shown, with the cleavage site in dotted line at -1/+1. Significant high and low values, in bold font are highlighted: grey: >10%; black: most preferred residue(s); cyan: charged residue group and green: aliphatic group.

**A. Eukaryote**	**-10**	**-9**	**-8**	**-7**	**-6**	**-5**	**-4**	**-3**	**-2**	**-1**	**+1**	**+2**	**+3**	**+4**	**+5**	**+6**	**+7**	**+8**	**+9**	**+10**
	**P10**	**P9**	**P8**	**P7**	**P6**	**P5**	**P4**	**P3**	**P2**	**P1**	**P1’**	**P2’**	**P3’**	**P4’**	**P5’**	**P6’**	**P7’**	**P8’**	**P9’**	**P10’**
Ala(A)	9.43	16.25	13.43	11.51	13.11	15.18	9.38	**25.84**	7.46	**48.91**	13.53	3.68	4.74	5.33	5.17	5.49	5.01	5.27	5.43	6.23
Cys(C)	3.68	4.05	5.91	5.81	3.68	2.18	3.36	6.50	1.49	3.57	3.41	1.92	7.73	4.16	3.46	5.01	4.85	3.84	3.84	3.89
Asp(D)	0.11	0.16	0.27	0.43	0.69	3.84	1.33	0.27	3.94	0.32	5.33	7.99	5.49	5.59	5.65	5.65	5.97	5.75	4.90	5.59
Glu(E)	0.32	0.32	0.85	1.39	0.69	2.02	2.66	0.37	8.79	0.53	7.83	8.84	5.22	7.19	7.25	6.45	6.02	6.71	5.97	6.07
Phe(F)	9.11	6.87	5.86	6.77	5.91	1.60	2.34	0.64	3.46	0.21	3.57	2.18	3.62	3.36	3.14	3.94	3.09	4.53	3.04	3.94
Gly(G)	4.00	3.94	5.27	4.32	3.46	11.13	13.96	9.43	3.52	**20.72**	7.14	5.01	6.29	8.10	6.66	7.19	5.91	8.15	8.95	8.58
His(H)	0.27	0.48	0.80	0.75	0.59	2.50	1.23	0.16	4.95	0.05	1.81	2.45	2.45	2.61	3.09	2.18	3.62	1.81	2.29	2.40
Ile(I)	5.17	6.18	4.37	4.05	7.94	2.29	3.41	3.78	1.70	0.16	3.30	3.73	6.23	2.72	3.14	3.46	3.14	3.57	4.79	3.52
Lys(K)	0.11	0.00	0.05	0.91	0.16	2.08	1.92	0.37	1.44	0.11	4.64	4.95	2.45	4.79	5.38	4.85	4.85	3.84	5.27	5.22
Leu(L)	**43.79**	**37.93**	**29.89**	**36.49**	**27.22**	7.94	16.41	4.32	15.24	1.39	8.47	4.58	9.11	5.65	5.97	7.46	6.55	7.67	8.26	7.25
Met(M)	2.40	1.97	3.30	2.50	1.81	1.17	1.70	0.27	1.76	0.21	1.12	0.69	1.70	1.28	1.70	1.17	1.33	2.13	1.44	1.70
Asn(N)	0.69	0.48	0.59	1.23	0.75	2.02	0.91	0.69	4.21	0.37	2.50	4.32	3.73	3.94	4.32	3.25	5.43	4.37	4.26	4.95
Pro(P)	1.01	1.17	0.96	2.34	6.29	9.38	9.11	0.21	0.69	2.02	0.27	15.82	7.46	10.66	8.79	8.58	8.68	9.86	6.93	6.55
Gln(Q)	0.64	0.75	1.97	1.65	1.07	5.27	4.48	0.27	7.25	1.33	11.03	4.90	3.52	6.77	5.54	5.54	5.06	3.84	6.93	4.48
Arg(R)	0.11	0.21	0.37	1.17	0.75	2.98	2.88	0.37	5.54	0.96	4.95	4.69	2.29	3.62	5.59	6.13	4.58	5.49	4.00	4.21
Ser(S)	3.30	6.23	7.88	6.29	6.93	12.20	8.47	13.00	11.99	13.48	8.20	9.00	7.62	7.94	7.94	6.55	6.87	8.15	8.31	6.61
Thr(T)	4.26	3.20	4.53	3.62	4.16	8.90	6.39	10.97	4.05	5.01	4.26	5.59	6.61	7.51	7.46	6.39	6.82	5.59	4.90	7.51
Val(V)	10.28	8.74	10.87	5.81	12.36	5.17	7.46	**22.32**	3.57	0.37	4.48	6.34	8.74	5.11	5.70	7.62	6.39	5.65	6.55	6.39
Trp(W)	0.91	0.64	1.76	2.13	1.65	1.17	1.23	0.05	4.21	0.16	1.01	0.85	1.86	0.80	0.69	0.75	1.07	1.65	1.01	1.76
Tyr(Y)	0.43	0.43	1.07	0.85	0.80	0.96	1.39	0.16	4.74	0.11	3.14	2.45	3.14	2.88	3.36	2.34	4.74	2.13	2.93	3.14
Charged	**0.91**	**1.17**	**2.34**	**4.64**	**2.88**	13.43	10.02	**1.55**	24.67	**1.97**	24.56	28.93	17.90	23.81	26.96	25.25	25.04	23.60	22.43	23.49
Small	16.73	26.43	26.58	22.11	23.49	38.52	31.81	48.27	22.96	**83.11**	28.88	17.69	18.65	21.36	19.77	19.23	17.79	21.58	22.70	21.42
Aliphatic	**68.67**	**69.10**	**58.55**	**57.86**	**60.63**	30.58	36.65	**56.26**	27.97	**50.83**	29.78	18.33	28.82	18.81	19.98	24.03	21.10	22.16	25.04	23.39
Hydrophobic	38.41	41.82	40.60	36.01	49.23	38.04	36.55	**53.49**	24.29	**52.16**	31.91	38.25	36.81	34.04	33.72	35.86	33.56	36.49	34.47	35.32
Polar un-charged	17.00	19.07	27.22	23.76	20.83	42.67	38.95	41.02	37.24	44.59	39.69	33.19	38.63	41.29	38.73	36.28	39.69	36.07	40.12	39.16

**B. Gram +ve**	**-10**	**-9**	**-8**	**-7**	**-6**	**-5**	**-4**	**-3**	**-2**	**-1**	**+1**	**+2**	**+3**	**+4**	**+5**	**+6**	**+7**	**+8**	**+9**	**+10**
	**P10**	**P9**	**P8**	**P7**	**P6**	**P5**	**P4**	**P3**	**P2**	**P1**	**P1'**	**P2'**	**P3'**	**P4'**	**P5'**	**P6'**	**P7'**	**P8'**	**P9'**	**P10'**
Ala(A)	**25.60**	16.67	14.88	11.90	19.05	7.14	10.12	**51.79**	11.31	**83.93**	**36.31**	9.52	7.14	6.55	11.90	7.74	10.71	10.71	10.12	5.95
Cys(C)	0.60	1.19	0.60	0.60	0.00	0.00	0.00	0.00	0.60	0.00	0.60	0.00	0.60	1.19	0.60	0.60	0.00	0.60	0.60	0.00
Asp(D)	0.00	0.60	0.60	1.19	2.38	2.98	4.17	0.00	2.38	0.00	11.31	10.71	7.14	6.55	9.52	7.14	8.33	7.14	8.33	7.14
Glu(E)	1.79	0.00	0.60	1.79	1.19	3.57	4.76	1.79	5.36	0.00	9.52	13.69	5.36	4.17	8.33	5.95	5.95	6.55	5.95	8.33
Phe(F)	7.74	9.52	4.76	7.74	1.79	1.19	0.60	1.19	11.90	0.00	2.38	1.19	3.57	2.38	3.57	4.17	1.79	0.00	1.79	2.98
Gly(G)	6.55	5.95	11.90	7.14	7.14	8.33	6.55	1.79	4.17	4.76	1.19	7.74	8.93	8.33	5.36	6.55	4.17	4.76	6.55	7.14
His(H)	0.00	0.00	0.60	1.19	1.79	0.60	1.19	0.00	6.55	0.00	1.19	1.79	0.60	1.19	1.19	0.60	1.19	1.19	1.79	1.19
Ile(I)	5.36	6.55	5.36	5.36	4.76	1.79	3.57	2.98	1.19	0.00	1.79	1.19	3.57	3.57	2.38	4.17	7.14	2.98	2.98	2.98
Lys(K)	0.00	0.00	0.60	0.60	2.38	4.17	5.36	0.60	8.93	2.38	5.36	1.19	13.10	6.55	5.95	5.36	7.14	7.14	8.93	7.14
Leu(L)	19.64	**20.83**	15.48	11.90	5.36	3.57	4.76	1.19	5.36	1.19	1.19	1.19	5.36	2.98	5.36	4.17	4.76	6.55	8.33	4.76
Met(M)	1.19	2.98	5.95	3.57	1.19	0.60	3.57	0.60	1.79	0.00	0.60	0.00	0.60	0.60	0.60	0.60	1.79	1.79	1.79	1.19
Asn(N)	2.38	2.98	1.79	4.76	1.79	7.74	5.36	0.00	4.76	0.00	2.98	4.17	5.95	7.14	9.52	5.95	4.17	8.93	5.36	6.55
Pro(P)	0.00	5.95	4.76	5.95	17.26	11.90	12.50	0.00	0.60	0.60	0.00	13.10	7.14	14.29	3.57	9.52	7.74	7.14	7.14	2.38
Gln(Q)	0.60	0.60	1.19	5.36	5.36	5.95	3.57	0.60	11.90	0.00	5.36	1.79	4.17	4.17	4.17	7.74	3.57	2.98	3.57	5.95
Arg(R)	1.19	0.00	0.00	0.00	0.60	1.19	1.19	1.79	2.38	1.19	1.79	0.60	2.38	1.19	1.19	1.19	2.38	1.79	1.19	4.17
Ser(S)	6.55	10.71	10.12	7.14	7.74	16.07	6.55	9.52	12.50	3.57	10.71	10.71	8.33	5.36	7.74	4.17	8.93	8.93	8.93	8.33
Thr(T)	7.74	4.76	10.71	10.71	13.10	16.07	16.67	2.38	1.79	1.19	2.38	14.29	7.74	14.29	8.93	10.12	11.31	8.93	7.74	9.52
Val(V)	11.90	9.52	7.74	11.90	6.55	6.55	8.33	**23.81**	3.57	1.19	4.17	5.95	6.55	7.14	3.57	9.52	6.55	7.74	5.95	9.52
Trp(W)	0.60	1.19	1.19	1.19	0.60	0.60	0.60	0.00	0.60	0.00	0.60	0.00	0.00	0.60	1.19	1.79	1.19	0.00	0.60	1.79
Tyr(Y)	0.60	0.00	1.19	0.00	0.00	0.00	0.60	0.00	2.38	0.00	0.60	1.19	1.79	1.79	5.36	2.98	1.19	4.17	2.38	2.98
Charged	**2.98**	**0.60**	**2.38**	**4.76**	**8.33**	12.50	16.67	**4.17**	25.60	**3.57**	29.17	27.98	28.57	19.64	26.19	20.24	25.00	23.81	26.19	27.98
Small	38.69	33.33	36.90	26.19	33.93	31.55	23.21	**63.10**	27.98	**92.26**	48.21	27.98	24.40	20.24	25.00	18.45	23.81	24.40	25.60	21.43
Aliphatic	**62.50**	**53.57**	43.45	41.07	35.71	19.05	26.79	**79.76**	21.43	**86.31**	43.45	17.86	22.62	20.24	23.21	25.60	29.17	27.98	27.38	23.21
Hydrophobic	**52.38**	**52.38**	45.24	48.21	**53.57**	33.93	44.64	**80.95**	39.88	**88.10**	**51.19**	32.14	41.67	41.67	32.74	42.86	44.05	37.50	39.29	33.93
Polar un-charged	25.00	26.19	37.50	35.71	35.12	**54.17**	39.29	14.29	38.10	**9.52**	23.81	39.88	37.50	42.26	41.67	38.10	33.33	39.29	35.12	40.48

C. Gram -ve	-10	-9	-8	-7	-6	-5	-4	-3	-2	-1	+1	+2	+3	+4	+5	+6	+7	+8	+9	+10
	**P10**	**P9**	**P8**	**P7**	**P6**	**P5**	**P4**	**P3**	**P2**	**P1**	**P1’**	**P2’**	**P3’**	**P4’**	**P5’**	**P6’**	**P7’**	**P8’**	**P9’**	**P10’**
Ala(A)	22.48	23.45	16.94	16.94	30.62	16.94	16.61	61.89	6.19	93.16	41.69	5.86	8.14	8.14	11.40	8.79	9.45	8.47	7.49	11.40
Cys(C)	1.95	1.95	3.58	0.65	1.30	0.98	0.65	0.65	0.00	0.00	0.00	0.00	1.30	0.00	0.65	0.65	0.00	0.98	0.65	1.95
Asp(D)	0.33	0.33	0.33	0.00	0.00	0.00	0.33	0.00	0.00	0.00	7.17	17.26	3.58	9.45	5.21	6.19	6.51	5.21	4.56	6.84
Glu(E)	0.65	0.00	0.65	0.00	0.65	0.65	0.33	0.00	0.65	0.00	6.19	16.94	4.56	5.86	8.14	8.14	6.51	5.54	6.19	5.54
Phe(F)	4.56	7.49	7.17	14.33	1.95	7.17	2.93	0.98	11.40	0.00	0.98	0.98	3.58	1.30	1.95	1.95	4.89	3.26	1.95	2.61
Gly(G)	6.51	3.91	14.98	6.84	4.23	17.26	5.21	0.98	0.98	2.93	6.19	6.51	7.49	6.51	5.54	8.14	6.51	12.38	10.42	6.84
His(H)	0.00	0.00	0.33	0.65	0.00	1.63	0.65	0.33	11.40	0.33	0.98	0.33	0.98	0.98	1.63	2.61	1.95	0.98	1.30	0.65
Ile(I)	4.89	4.89	2.28	7.17	2.61	0.33	1.95	1.30	1.95	0.00	0.33	2.93	4.56	5.21	6.51	4.56	6.51	6.19	6.19	5.21
Lys(K)	0.00	0.33	0.00	0.33	0.00	0.33	0.65	0.00	0.98	0.00	2.93	1.63	6.19	3.58	2.93	8.14	4.23	7.17	6.51	10.75
Leu(L)	26.71	30.29	28.01	19.54	2.93	9.45	3.58	5.86	17.59	0.33	3.26	1.63	8.14	7.82	6.84	5.21	4.89	7.17	5.54	4.89
Met(M)	3.26	3.58	3.58	5.21	3.91	3.91	1.30	0.00	6.19	0.00	0.00	0.00	1.30	0.98	1.30	0.98	3.26	0.98	1.30	1.63
Asn(N)	0.65	0.65	0.00	0.98	0.98	3.26	4.56	0.33	4.56	0.33	3.58	5.86	5.21	6.19	9.45	6.19	4.23	3.91	8.47	4.89
Pro(P)	0.98	0.98	0.33	1.30	6.51	5.21	16.61	0.00	0.33	0.00	0.00	10.75	7.82	7.17	7.82	8.14	4.56	3.58	6.51	2.61
Gln(Q)	0.98	1.30	0.33	0.33	0.33	3.91	5.54	0.65	14.33	1.30	12.05	4.89	7.49	3.58	4.56	3.26	6.19	7.82	5.21	4.23
Arg(R)	0.00	0.65	0.65	0.33	0.00	0.98	0.65	0.98	0.98	0.00	0.65	0.33	0.98	2.28	0.65	2.93	3.58	1.95	0.98	1.95
Ser(S)	11.73	7.82	7.49	12.05	33.22	14.33	24.10	9.12	5.54	1.30	4.23	5.54	5.21	5.86	5.86	4.23	4.56	5.21	7.82	8.79
Thr(T)	4.89	4.23	5.86	4.89	5.86	8.47	6.84	4.56	3.91	0.33	3.91	10.75	11.07	13.03	6.19	9.77	6.84	7.49	7.49	6.84
Val(V)	7.82	7.49	6.84	6.19	4.23	4.56	4.56	12.38	5.86	0.00	3.26	5.86	9.77	6.51	9.45	6.84	10.10	6.84	6.19	8.47
Trp(W)	1.30	0.33	0.65	0.98	0.33	0.65	0.00	0.00	2.28	0.00	0.65	1.63	0.65	1.95	0.33	0.98	1.63	1.30	1.30	1.30
Tyr(Y)	0.33	0.33	0.00	1.30	0.33	0.00	2.93	0.00	4.89	0.00	1.95	0.33	1.95	3.58	3.58	2.28	3.58	3.58	3.91	2.61
Charged	**0.98**	**1.30**	**1.95**	**1.30**	**0.65**	**3.58**	**2.61**	**1.30**	14.01	**0.33**	17.92	36.48	16.29	22.15	18.57	28.01	22.80	20.85	19.54	25.73
Small	40.72	35.18	39.41	35.83	**68.08**	48.53	45.93	**71.99**	12.70	**97.39**	**52.12**	17.92	20.85	20.52	22.80	21.17	20.52	26.06	25.73	27.04
Aliphatic	**61.89**	**66.12**	**54.07**	49.84	40.39	31.27	26.71	**81.43**	31.60	**93.49**	48.53	16.29	30.62	27.69	34.20	25.41	30.94	28.66	25.41	29.97
Hydrophobic	45.28	48.53	37.79	**52.44**	**50.16**	39.09	44.63	**76.55**	35.18	**93.16**	49.84	29.64	42.02	34.85	41.69	40.39	44.63	37.79	37.46	43.97
Polar un-charged	27.04	20.20	32.25	27.04	46.25	48.21	49.84	16.29	34.20	**6.19**	31.92	33.88	39.74	38.76	35.83	34.53	31.92	41.37	43.97	36.16
